# An assessment of staffing needs at a HIV clinic in a Western Kenya using the WHO workload indicators of staffing need WISN, 2011

**DOI:** 10.1186/s12960-017-0186-3

**Published:** 2017-01-26

**Authors:** B. Burmen, N. Owuor, P. Mitei

**Affiliations:** 1 Kenya Medical Research Institute Center for Global Health Research, P.O. Box 1578-40100, Kisumu, Kenya; 2 Jaramogi Oginga Odinga Teaching and Referral Hospital, BOX 849-40100, Kisumu, Kenya

**Keywords:** Adequacy of human resources, Staffing needs, HIV/AIDS health services

## Abstract

**Background:**

An optimal number of health workers, who are appropriately allocated across different occupations and geographical regions, are required to ensure population coverage of health interventions. Health worker shortages in HIV care provision are highest in areas that are worst hit by the HIV epidemic. Kenya is listed among countries that experience health worker shortages (<2.5 health workers per 1000 population) and have a high HIV burden (HIV prevalence 5.6 with 15.2% in Nyanza province). We set out to determine the optimum number of clinicians required to provide quality consultancy HIV care services at the Jaramogi Oginga Odinga Teaching and Referral Hospital, JOOTRH, HIV Clinic, the premier HIV clinic in Nyanza province with a cumulative client enrolment of PLHIV of over 20,000 persons.

**Case presentation:**

The World Health’s Organization’s Workload Indicators of Staffing Needs (WISN) was used to compute the staffing needs and sufficiency of staffing needs at the JOOTRH HIV clinic in Kisumu, Kenya, between January and December 2011. All people living with HIV (PLHIV) who received HIV care services at the HIV clinic at JOOTRH and all the clinicians attending to them were included in this analysis. The actual staffing was divided by the optimal staff requirement to give ratios of staffing excesses or shortages. A ratio of 1.0 indicated optimal staffing, less than 1.0 indicated suboptimal staffing, and more than 1 indicated supra optimal staffing. The HIV clinic is served by 56 staff of various cadres. Clinicians (doctors and clinical officers) comprise approximately one fifth of this population (*n* = 12). All clinicians (excluding the clinic manager, who is engaged in administrative duties and supervisory roles that consumes approximately one third of his time) provide full-time consultancy services. To operate at maximum efficiency, the clinic therefore requires 19 clinicians. The clinic therefore operates with only 60% of its staffing requirements.

**Conclusions:**

Our assessment revealed a severe shortage of clinicians providing consultation services at the HIV clinic. Human resources managers should oversee the rational planning, training, retention, and management of human resources for health using the WISN which is an objective and reliable means of estimating staffing needs.

## Background

A health worker density exceeding 2.5 per 1000 population (or 23 health workers—doctors, nurses, and midwives—per 10,000 populations) is required to achieve the Sustainable Development Goal (SDG) of ensuring healthy lives and promoting well-being for all at all ages [1]. However, there is a global health worker shortage in 57 developing countries, 36 of which are in Africa [2]. With the advent and scale-up of antiretroviral therapy (ART), and drastic reductions in the costs of ART, there has been a decline in morbidity and mortality due to HIV. However, health worker shortages continue to pose a formidable challenge in ART provision. HIV programs in countries with the highest HIV burden in 2004 (Malawi, Zimbabwe, South Africa, and Mozambique) continue to cite health worker shortage as a major impediment to achieving their goals [3, 4].

Kenya, with a population of 40 million and a HIV prevalence of 6.2% among persons aged 15–49 years of age, had only attained 13 of the minimum requirement of 23 health workers per 10,000 populations in 2010 [5]. Nyanza province of Kenya had the highest HIV prevalence in the country: 15.6% against the country’s average of 5.1% according to the Kenya AIDS Indicator Survey of 2012 [6].

To address the global health workforce crisis, tactical information on human resources for health should be provided to guide policy making [2]. This implies that we need information to forecast the number of health workers required. For this reason, the Kenya National AIDS strategic plan III 2009–2013 directed that adequacy of human resources for health be assessed using staff audits [7].

To compute optimal allocations and deployment of staff, population ratios, standard staffing schedules, or the Workload Indicators of Staffing Needs (WISN) can be used. Population ratios are based on the World Health Organization (WHO) recommendations of number of health workers per a catchment population. However, population ratios do not consider that workloads may differ in different localities; hospitals with the same bed capacity may not have same morbidities, health seeking habits, and facility workloads. Additionally, health requirements will vary with population density, demographic and socioeconomic characteristics, morbidity and mortality, geographical features, utilization patterns, and ease of access [8]. Standard staffing schedules, similar to benchmarking, are based on a predetermined number of health workers who should be attached to a given hospital [9]. Fixed staffing norms in health facilities in Uganda have been shown to fall short of WISN staffing requirements [10]. Majority of staffing norms are usually located somewhere between the two. Subsequently, unadjusted staff loads lead to overstating staffing needs or underestimating workloads [9, 11]. Other methods to assess staffing needs include using informal managerial judgments [12].

An ideal method would be to use the WISN, developed by the WHO. The WISN includes activities done by common cadres, the annual workload, time taken to do particular activities, available working time, and associated activities that are not core to the job description of a person [11]. WISN takes into account the indigenous epidemiology and specific sets of services; therefore, its results are precise and more useful for planning and policy implementation [13]. WISN carried out to assess health worker requirements in Indonesia, India, Bukina Faso, Namibia, Mozambique, and Uganda have illustrated staffing excesses, shortages, or adequacy [8–10, 13–15]. The Jaramogi Oginga Odinga Teaching and Referral Hospital (JOOTRH, formerly New Nyanza provincial General Hospital) is Nyanza province’s largest regional referral public health facility. We assessed the staffing needs and the sufficiency of staffing needs of persons providing consultancy services at JOOTRH HIV clinic, a busy clinic in a high HIV burden area using the WISN.

## Case presentation

### Study site and setting

A case study was conducted at the Jaramogi Oginga Odinga Teaching and Referral Hospital (JOOTRH) in Kisumu, Kenya, a referral facility in the former Nyanza Province and accessible to Western Province and part of Rift valley Province in Kenya. It serves a population in excess of 5 million people and has a bed capacity of 467 with occupancy of approximately 95% [16]. The JOOTRH is the province’s largest regional referral public health facility. It was the premier facility to provide HIV health services in the province, and it has the largest number of PLHIV ever and currently enrolled in the province. The JOOTRH HIV clinic was initiated in 2003. Up to 2011, there was a cumulative enrollment of 21,000 PLHIV with 10,000 of them currently active and on care. Other PLHIV had either been transferred to other HIV clinics during the HIV program scale-up and patient decentralization as other health facilities begun providing ART, or were lost to follow-up, or had died. For these reasons, JOOTRH was a suitable location for the evaluation of HRH capacities in HIV care and treatment.

### Study population

All people living with HIV (PLHIV) who received HIV care services at the HIV clinic at JOOTRH between January and December 2011 (*n* = 10,000) and all the clinicians (*n* = 10) attending to them on a full-time basis were included in the analysis. At the time of this evaluation, only clinicians (doctors and clinical officers) were providing consultancy services at this clinic. No subjects (PLHIV or clinicians) were excluded from the sample.

### Scheduling of clinic appointments

A HIV-infected individual was defined as “active on care” if he or she had honored all their clinic appointments up to 3 months preceding the analysis date. All newly diagnosed HIV-infected persons who were enrolled at the HIV clinic were reviewed at the first clinical visit and baseline laboratory and radiological investigations were done, then 2 weeks later to review the results of the baseline laboratory tests, then 2 weeks later for initiation of ART if eligible for ART. After this, PLHIV would be reviewed monthly or quarterly, depending on their medical condition. This translates to 8 to 12 visits in a year. However, the number of visits may exceed 12 if the individual’s medical condition warrants it. The number of clinical visits required for PLHIV is summarized in Table 1 [17].

**Table 1:** Summary of clinical and laboratory follow-up for HIV-infected patients on ART

	Week		Month							
Appointment	0	2	1	2	3	4	5	6	8	12
Clinical evaluation	†	†	†	†	†	†	†	†	†	†
TB screening	†	†	†	†	†	†	†	†	†	†
Adherence check	†	†	†	†	†	†	†	†	†	†
Hb	†		†		†	Symptom directed				
ALT	†		†		†	Symptom directed				
Creatinine	†	Symptom directed								
Pregnancy test	†	If indicated								
Urinalysis	†	Symptom directed								
Fasting lipid profile and glucose	†	Annually for patients on PIs								
CD4 count	†							†		†
Viral load		Targeted								

Source: National AIDS and STI Control Program N. Guidelines for antiretroviral therapy in Kenya 4th Edition. Nairobi: Ministry of Medical Services [17]

† Specific investigation was required at that visit

### Data collection

Quantitative and qualitative data was collected by two of three of the authors using key informant interviews, review of source documents, e.g., the National guidelines for ART management [18], quarterly quality of care reports at the facility, and labor laws and human resource policies in Kenya [19] Qualitative data was collected from two key informants (clinicians) at JOOTRH using a checklist adapted from the WISN. We conducted document review to obtain quantitative information on hospital service statistics from hospital records, HIV management guidelines, and workload components. The average time it took to complete one consultation was established by observing consultations with time check, for 50 PLHIV on different days at the clinic.

### Data analysis

The WHO’s WISN was used to compute the staff excesses or shortages at the HIV clinic.

The WISN is based on estimating the time taken to do particular activities by common cadres, the annual workload, available working time per staff, and associated activities that are not core to the job description of a person.

A department was selected, then the core activities done in that department, and the standard time required to complete those activities to satisfaction were established by observation.

The annual workload was then ascertained by finding out how many of the core activities had to be performed annually.

The annual available working time per staff member was then computed by calculating all the working days in a year (excluding the 10 public holidays in Kenya and the 30 days of annual leave and the time spent on weekly meetings) [19].

The baseline staffing requirement was then computed by dividing the annual workload by the annual available working time per staff member. This baseline staffing requirement, however, did not factor in the category allowance. The category allowance is the proportion of time spent by an individual doing activities done outside his/her regular job description during working hours that would reduce his annual available working time.

The category allowance was then used to compute the multiplier quotient (the reciprocal of the category allowance).

The intermediate staff requirement, which allows for the inclusion of the category allowance in computing the staffing requirements, was given by multiplying the baseline staff requirements by the multiplier quotient.

The intermediate staff requirements, however, did not consider the amount of time spent by the departmental head attending to managerial duties (which reduced the time he spent on consultations). This time was referred to as the individual allowance. The intermediate staff requirements did not also reflect the shift allowance which was time off given to staff for working nights, on weekends or public holidays. The shift allowance, however, is not included for the departments that operate only during regular working hours, i.e., from 8.00 am to 5.00 pm (including a 1-h lunch break).

The individual allowance was calculated by finding out the proportion of annual available working time spent on non-routine activities, e.g., attending senior management meetings, supervision, training interns, and other staff, hospital medical therapeutics meeting, hospital HIV committee meeting, hospital ERC meeting every, ad hoc meetings, and courtesy calls—administrative issues.

The final staff requirements were then computed by adding the baseline staffing requirements to the individual allowance.

Staffing excesses or shortages were computed by subtracting the final staff requirements from the actual staffing in that department. A negative figure indicated suboptimal staffing (staff shortages) and a positive figure indicated supra optimal staffing (staff excesses). A figure of zero indicated adequate staffing requirements. Staffing excesses or shortages were also expressed as ratios; the actual staffing was divided by the final staff requirement. A ratio of 1.0 indicated optimal/adequate staffing, less than 1.0 indicated a staff shortage and more than 1 indicated a staffing excess [20].

### Ethical considerations

Ethical approval was sought and obtained from the Kenya Medical Research Institute Ethical Review Board (SSC 1525).

### Department selected

The consultation clinics in the HIV clinic were selected for estimation of staffing needs using WISN.

### Composition of staff at the JOOTRH HIV clinic 2011

The HIV clinic is served by 56 staff of various cadres: 12 (22%) are clinicians (doctors and clinical officers) who provide consultation services (Table 2).

**Table 2:** Composition of staff at the JOOTRH HIV clinic 2011

Cadre	Positions occupied *n* (%)
Nursing staff	11 (20%)
Clinical officers	10 (18%)
Peer educators	7 (13%)
Counselors	6 (11%)
Clinic assistants	5 (9%)
Data clerks	5 (9%)
Data officers	4 (5%)
Data assistants	3 (3%)
Doctors	2 (4%)
Support staff/cleaners	2 (4%)
Nutritionist	1 (2%)
Total	56

### Staffing requirements at the HIV clinic

Overall, 3125 working days are required to provide adequate consultancy services to the 10,000 HIV-infected patients seen at the HIV clinic. With only 179 working days available to each clinician, 17.46 clinicians are required to run this clinic on a full-time basis. The services of one other clinician will be required in addition to the computed 17.46 should all the clinicians participate in the required 10-day training sessions annually (Table 3).

**Table 3:** Computation of estimating staffing requirements using WISN

Establish activity and its standard
The activity chosen for the clinicians who run the consultation clinic was a consultation which is estimated to last 15 min (0.25 h).
Ascertain the workload (sum activity standards)
Each patient was estimated to have up to 10 scheduled clinic appointments in a year (Table 1). For 10,000 patients, this translates to 100,000 clinic appointments. With each appointment lasting 0.25 h, the annual workload would require 25,000 h to complete. With each working day lasting 8 h, this translated to 3125 days.
Establish annual available working time
This was the number of days spent in consultations each week. Clinicians spend 4.5 days a week in consultations and 0.5 days a week in a multidisciplinary team meeting. There are 52 weeks in a year so this translates to 234 days in a year spent on consultations. There are 10 public holidays in Kenya when the clinic remains closed and this reduces the annual available working time to 224 days. Each staff member is entitled to 30 days of annual leave which reduced the annual available working time to 194 days. Each staff member is also allowed 15 days of sick leave on full pay which effectively reduces the annual available working time per clinician to 179 days.
The baseline staff requirement (divide workload by sum activity standards)
The baseline staff requirement was given by the annual workload (3125 days) divided by the annual available working time (179 days) = 17.46 staff
Calculate category allowance and multiplier quotient
The category allowance, the time a clinician spent on non-clinical activities (training sessions) each year was ascertained to be 10 days. The proportion of annual available working time spent on non-clinical duties (10/179 = 0.056).
The multiplier quotient is the reciprocal of 1 − category allowance. The multiplier quotient = 1/(1 − category allowance) =1/(1 − 0.056) =1.06
The intermediate staff requirement
The intermediate staff requirement = the baseline staff requirement (17.46) × the multiplier quotient (1.06) = 18.51
Calculate individual allowance
The lead clinician spends 4.375 days monthly on non-clinical duties (the individual allowance as shown in Table 4). The individual allowance is therefore 4.375 × 12/179 = 0.29
Optimal number of clinical staff Optimal number of clinical staff = intermediate staff requirements + individual allowance = 18.51 + 0.29 = 18.8~19 clinicians
Staffing requirements Number of clinicians available = 12 Staff shortage = optimal number of clinical staff-number of clinical staff available = 7 clinicians Current staff proportion = number of clinical staff available/optimal number of clinical staff =12/19 =0.63 of the optimal staff requirements

This computation assumes that all the clinicians provide consultancy services on a full-time basis. However, the clinic manager, who is also a clinician, is also engaged in administrative duties and supervisory roles which effectively reduce the amount of time he spends seeing patients. This consumes approximately one third of his time necessitating an additional 0.3 clinician to take up consultancy services when he is away (Table 4).

**Table 4:** The individual allowance of the clinic manager

Activity	Duration in hours	Monthly frequency	Total monthly duration in hours
HIV program coordinators’ meeting	2	4	8
Hospital HIV committee meeting	2	1	2
Hospital ERC meeting	2	2	4
Ad hoc meetings	1	2	2
Courtesy calls	1	4	4
Hospital medical therapeutics	3	1	3
Administration	3	4	12
Total individual allowance per month in hours			35
Total individual allowance per month in days (1 day = 8 h) = 4.375			
Total annual individual allowance per year in days = 4.375 × 12 = 52.5 days			

The optimal staffing requirements were given by the sum of the intermediate staffing requirements and the individual allowance which was 19 (Table 3). In summary, this clinic requires 19 clinicians to operate at maximum efficiency.

Steps in estimating staffing requirements using WISN are summarized in Table 4.

### Assessing the sufficiency of human resources for health at the HIV consultation clinic

This clinic is served by 12 clinicians (Table 2). This clinic therefore experiences a staff shortage of 7 clinicians and is operating with only 60% of its staffing requirements (Table 3).

### Discussion

To the best of our knowledge, this is the first evaluation that has estimated the staffing requirements relative to the patient population in HIV care services in Kenya. We found a shortage of clinical staff to provide consultation services at the HIV clinic at the JOOTRH, and a considerable amount of time was spent by the clinic manager on non-clinical duties.

The recommended number of clinicians to provide consultancy services at this clinic is similar to the 1–2 physicians per 1000 patients established in a review of published literature to estimate the health workforce needs for ART in resource-limited settings [4]. With only 60% of the optimal number of clinicians working at the clinic, it is expected that the consultation time will be considerably reduced. A separate patient flow analysis at the same clinic showed that an actual consultation takes only 3–5 min with 90% of the time patients spend in hospital being spent waiting [21]. WISN assessments that were done in Mozambique similarly illustrated an insufficiency of health workers of all cadres implying that all services were rushed and therefore compromising the quality of care [14, 20].

Different cadres currently propose minimum requirements for annual continuous medical education sessions for licensure to ensure that their members are up to date with the latest developments in the field [22]. New research also mandates changes in HIV care. Clinicians are therefore tasked with ensuring they are up to date with their licensing requirements as well as with knowledge on patients care. Additional minimum packages of training requirements have been proposed by HIV programs. Clinicians may therefore spend more than the allocated 10 days a year in training which has an impact on the available time spent on clinical services. Additionally, with evolution of science and availability of emerging evidence, guidelines for clinical care are also adapted. This may impact the clinicians’ available working time because these adjustments are made with no commensurate adjustments in the number of health workers as was seen in a Namibian assessment of staffing needs [23].

Quality of care indicators have traditionally focused on the quality of clinical care, e.g., the proportion of eligible patients in receipt of particular services without focusing on the time it takes to provide such a service [24]. Allocating the appropriate time to service provision may ensure that the required proportion of patients is in receipt of essential services. Quality care means that PLHIV obtain the care they require to maintain their health and quality of life and HIV programs and policy makers optimize the use of their resources [25]. If quality HIV care is to reach all those in need, significant improvement in staffing levels for clinicians are required. However, with the global health worker shortage and time it takes to train a clinician, alternatives like task shifting, which have been implemented with varying levels of success elsewhere, should be considered [26]. Task shifting effectively increases the number of health workers to perform a specific duty and maximizes the use of already limited number of health workers. Indeed, even at this clinic, an express nurse desk was later implemented for consultations with stable patients in need of a replenishment of their ART prescriptions only [21]. Later in 2014, the Nurse Initiated Management of antiretroviral Treatment (NIMART) was introduced by Columbia University in selected health facilities in Kenya to promote nurse initiation and management of ART [27].

In the literature, the application of WISN has illustrated inefficiency in time allocated to non-clinical duties by clinical staff. HIV programs could also optimize the use of clinicians by deploying them for critical and technical duties only [28]. For example, business managers or office administrators could be hired to assist with non-clinical managerial duties of clinics (with different proponents, and opponents) are proposed to free up clinicians’ time to attend to patients especially in areas with extreme health worker shortages. This may be a suitable substitute considering that the number of hours spent by the manager on managerial duties may increase when managers have to attend meetings outside the hospital, e.g., stakeholders’ meetings, trainings on new updates, and review of data collection tools. WISN assessments done in Indonesia illustrated that as opposed to a need to increase the number of midwives, there was a need to ensure midwives, who spent up to 50% of their time on other duties, were engaged in their core activities. The assessment also revealed that there was no need for extra midwives [14].

The application of WISN has also illustrated excesses in staffing levels. In an assessment in Uganda, one facility had a higher number of staff (other than medical officers) than those required to perform core and support activities. Similarly, in Bukina Faso, at the time of the evaluation, the current staff was adequate to handle the workload at the maternity [15, 28]. HIV programs could also consider equitable distribution of clinical workers from areas of excesses to areas of deficit [15].

#### Limitations

Our evaluation was not without limitations. WISN based on the annual workload which varies during the year and is unlikely to remain constant, e.g., there may be patient attrition or patients may need a longer consultation and may also have non-scheduled appointments. The number of visits may also exceed 12 if the patient’s medical condition warrants it. The expanded role of nurse-initiated ART management is not yet uniformly distributed in Kenya. This has an implication on the number of “clinicians” providing consultancy services at HIV clinics. The activity standard, i.e., the time required for an actual consultation, may have also been inaccurate [8].

We did not factor in a maternity leave allowance for the female staff. With the labor laws in Kenya revised to provide 90 calendar days for female staff for maternity leave [29] (a welcome change for career women), employers computation need to inculcate the number of female staff of reproductive age in their workforce, the fertility rate, and viability indicators when computing staffing needs. We were also unable to factor in possibilities or resignations or emergency leave.

## Conclusions

Our assessment revealed a severe shortage of clinicians providing consultation services at the HIV clinic negatively impacting the quality of clinical care provided. There was a possibility of these staffing deficits being compounded by the extra time required by the clinicians to attend training sessions, to take maternity leave or by staff resignations or sick leave or by the clinic manager spending time on non-clinical duties. Nevertheless, basic clinical care tasks could be shifted to other health workers, e.g., nurses to offset the shortage in the number of clinicians and ensuring that time spent by clinicians on non-clinical duties is minimized [14, 25].

There is need for an audit to assess the staffing requirements for all cadres of health workers in health systems using the minimum package of health as the activity standard [30]. The minimum package of health was able to illustrate the magnitude of staffing required to meet the service guarantees of India’s National Rural Health Mission [13]. With the changing guidelines for ART management in 2015 (Test and Treat), there will be a huge demand for ART services for PLHIV that range from those that are clinically stable to those with advanced HIV disease.

These populations will require different modes of HIV service delivery, and HIV programs should optimally allocate its human resource to serve these diverse needs. Alternative forms of ART delivery, e.g., community ART delivery, task shifting to ease burden of health facilities that are currently providing 95% of ART, could be employed. Similar WISN assessments, initially to assess staffing requirements, then later to establish the optimum number of clinicians to provide consultancy services at ART clinics and subsequently to forecast staffing needs, should then be repeated incorporating nurse-prescribers from the recently launched nurse-managed ART program (NIMART) [27, 31]. With this information, human resources managers can then oversee the planning, training, retention, and management of human resources for health to counteract staff deficits in the long term. Employment in the ministry of health should be driven by rational allocation of health workers of different cadres driven by WISN which is an objective and reliable means of estimating staffing needs [11].

## Abbreviations

AIDS: Acquired immune deficiency syndrome
ART: Antiretroviral therapy
HIV: Human immunodeficiency virus
MDG: Millennium Development Goals
NIMART: Nurse Initiated Management of antiretroviral Treatment
SDG: Sustainable Development Goal
SSC: Scientific Steering Committee
WHO: World Health Organization
